# Sensitivity to TOP2 Targeting Chemotherapeutics Is Regulated by Oct1 and FILIP1L

**DOI:** 10.1371/journal.pone.0042921

**Published:** 2012-08-10

**Authors:** Huarui Lu, Timothy C. Hallstrom

**Affiliations:** Department of Pediatrics, University of Minnesota, Minneapolis, Minnesota, United States of America; North Carolina State University, United States of America

## Abstract

Topoisomerase II (TOP2) targeting drugs like doxorubicin and etoposide are frontline chemotherapeutics for a wide variety of solid and hematological malignancies, including breast and ovarian adenocarcinomas, lung cancers, soft tissue sarcomas, leukemias and lymphomas. These agents cause a block in DNA replication leading to a pronounced DNA damage response and initiation of apoptotic programs. Resistance to these agents is common, however, and elucidation of the mechanisms causing resistance to therapy could shed light on strategies to reduce the frequency of ineffective treatments. To explore these mechanisms, we utilized an unbiased shRNA screen to identify genes that regulate cell death in response to doxorubicin treatment. We identified the Filamin A interacting protein 1-like (FILIP1L) gene as a crucial mediator of apoptosis triggered by doxorubicin. FILIP1L shares significant similarity with bacterial SbcC, an ATPase involved in DNA repair. FILIP1L was originally described as DOC1, or “down-regulated in ovarian cancer” and has since been shown to be downregulated in a wide variety of human tumors. FILIP1L levels increase markedly through transcriptional mechanisms following treatment with doxorubicin and other TOP2 poisons, including etoposide and mitoxantrone, but not by the TOP2 catalytic inhibitors merbarone or dexrazoxane (ICRF187), or by UV irradiation. This induction requires the action of the OCT1 transcription factor, which relocalizes to the FILIP1L promoter and facilitates its expression following doxorubicin treatment. Our findings suggest that the FILIP1L expression status in tumors may influence the response to anti-TOP2 chemotherapeutics.

## Introduction

The efficacy of cancer chemotherapy is influenced by numerous factors, including acquired mutations within the tumor and also by polymorphisms present in a patient. A frequent cause of chemotherapeutic resistance occurs through the amplification of ATP-binding cassette (ABC)-transporter proteins like the multidrug resistance proteins (Mdr1/P-glycoprotein or Mrp) [1], [2]. These proteins act as efflux pumps for a wide variety of structurally and chemically unrelated substrates [3]. Therapies that target specific proteins, such as the inhibition of epidermal growth factor receptor (EGFR) by gefitinib and erlotinib, can be disrupted by amino acid substitutions in EGFR that maintain protein functionality but lead to evasion of small molecule inhibitor interactions [4], [5]. Similarly, point mutations occurring in BCR-ABL cause its evasion of imatinib treatment [6]. Other studies report that activation of oncogenic signaling pathways like PI3K/Akt, or loss of tumor suppressor genes like p 53 confer resistance to chemotherapeutic agents [7], [8]. It also seems likely that the efficacy of drugs that induce cell death by different mechanisms, for example by DNA damage vs. microtubule stabilization, will be affected by different types of mutations. Given the huge obstacle that drug resistance poses for cancer therapy, it is critical to identify and characterize other mechanisms that alter chemotherapy efficacy.

Doxorubicin (Adriamycin) is a DNA intercalating agent that inhibits topoisomerase II function during DNA replication, causing DNA damage that kills the affected cell [9]. It is used as frontline treatment for many types of solid tumors, hematological malignancies and soft tissue sarcomas. However, some tumors appear innately resistant, while others become resistance to chemotherapy through acquired mutations, sometimes with mutations directly within topoisomerase subunits [10]. Such resistance is a major obstacle to successful therapy, as tumors that initially show a response can recur and are refractory to additional treatment with identical regimens.

RNA interference (RNAi) is a cellular process that silences specific genes through RNA induced silencing complex (RISC) dependent double-stranded RNA recognition and degradation [11]. This process can be used to cleave specific endogenous RNAs by exogenously adding virus or plasmid that expresses the reverse RNA template. Genome-wide libraries of such knock-down plasmids allow forward genetic screens to be performed in a variety of cells [12]. A previous shRNA screen looking at mediators of doxorubicin resistance identified TOP2A as a determinant of drug response [13]. We screened an unbiased library to identify other genes that potentially contribute toward cellular doxorubicin resistance.

In this work we identified Filamin A interacting protein 1-like (FILIP1L) as a potential mediator of doxorubicin induced apoptosis. The FILIP1L gene is highly induced by doxorubicin treatment and by other Topoisomerase II (TOP2) poisons like etoposide and mitoxantrone but not by the TOP2 catalytic inhibitors merbarone or dexrazoxane (ICRF187). Furthermore, expression of FILIP1L requires the activities of ATM/ATR and also the transcription factor Oct1. Doxorubicin treatment causes recruitment of the Oct1 transcription factor to the FILIP1L promoter. Our results indicate that doxorubicin induction of FILIP1L leads to cell death and that this potential mechanism of resistance should be further explored in tumor cells. Furthermore, since FILIP1L expression is lost in a variety of human tumor types, a correlation between reduced expression levels of FILIP1L and impaired response to doxorubicin chemotherapy should be explored.

## Results

**Figure 1:**
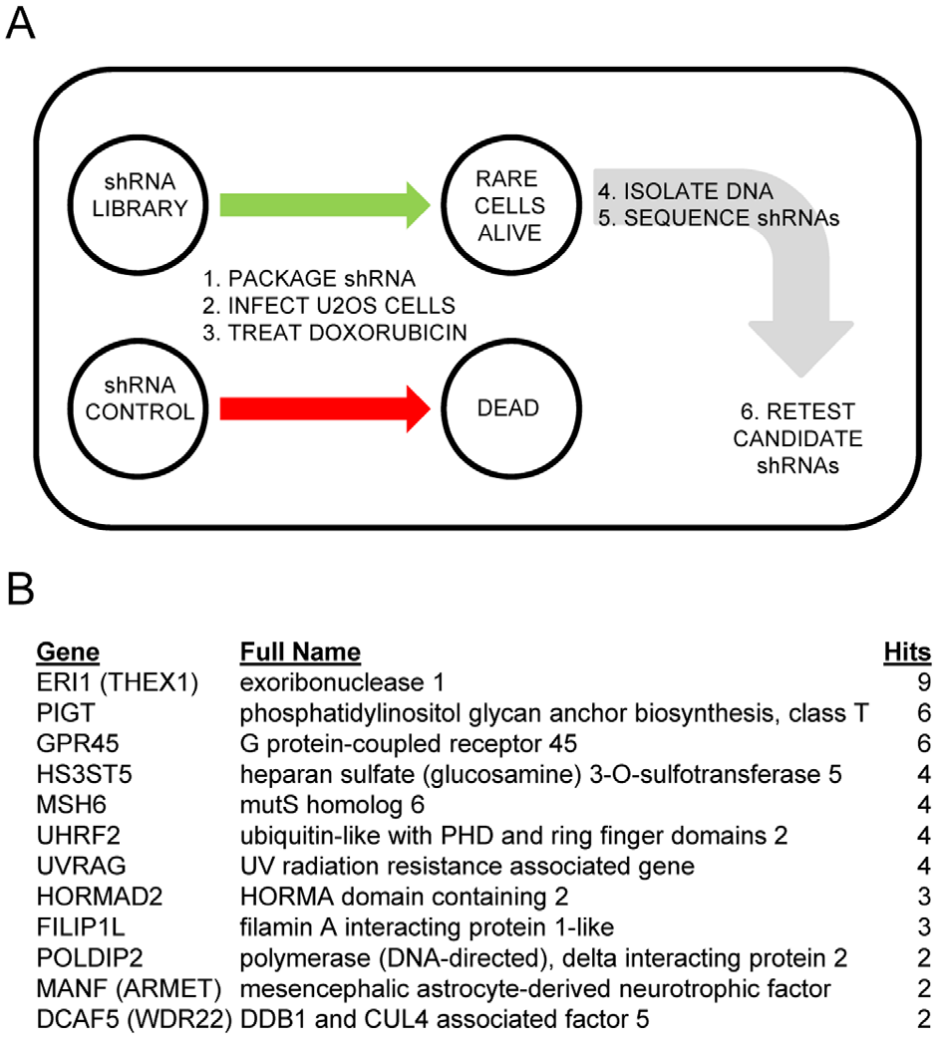
A functional shRNA screen for regulators of doxorubicin induced apoptosis. (A) Outline of the doxorubicin induced apoptosis bypass screen using U2OS cells. Pools of shRNA were transfected into retroviral packaging cell lines, and retrovirus transduced into U2OS cells followed by puromycin selection. Transduced U2OS cells were treated with 225 ng/ml Doxorubicin for 5 days, which led to apoptotic death of approximately 99.8% of the library infected cells. We harvested cells that survived treatment, isolated genomic DNA, PCR amplified the region containing shRNA sequences, shotgun cloned and sequenced. A total of approximately 1500 inserts were sequenced. (B) Twelve genes identified by this screen are listed. Full gene names and the number of times identified are also listed.

We used a pooled shRNA screening approach to identify genes that impair doxorubicin induced apoptosis when knocked down (Figure 1A). We determined levels of doxorubicin required to induce apoptosis in U2OS cell by administering increasing dosages and observing effects on cell death. We determined that 225 ng/ml doxorubicin killed 100% of plated control cells after 5 days. We reasoned that shRNAs that conferred resistance to doxorubicin mediated killing would essentially lower cells below this killing “threshold”, allowing them to survive treatment and be identified. Following doxorubicin treatment of shRNA integrated cells, rare surviving cells were observed. These doxorubicin resistant cells were trypsinized, pooled, and genomic DNA recovered from them. We PCR amplified the region of the plasmid containing the shRNA insert, cloned and sequenced products. We sequenced approximately 1500 clones and have listed recurring clones in Figure 1B.

**Figure 2:**
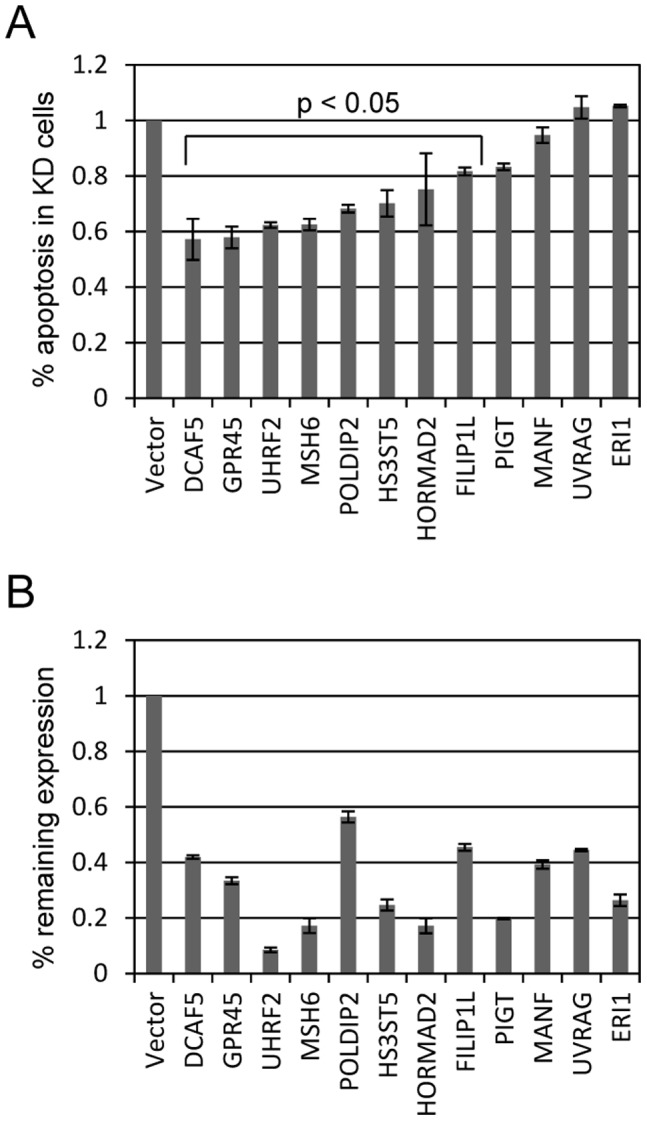
Identification of mediators of doxorubicin induced apoptosis. To determine which genes identified by our screen were true or false positives, we targeted each for degradation by shRNA. (A) Individual genes listed in 1B were targeted for shRNA mediated degradation in U2OS cells. shRNA targeted and control cells were treated with 400 ng/ml doxorubicin and measured by propidium iodide (PI) assay 72 hours later. Levels of apoptosis are reported as % apoptosis in shRNA targeted cell compared with vector control cells. Five of the cell lines appeared to be false positives and did not display reduced doxorubicin induced apoptosis. The other lines were impaired by 20–40% for doxorubicin induced apoptosis. (B) Knockdown levels in these cell lines were determined by qPCR comparing with vector control cells and listed as % remaining expression in target cells in 2A. Genes are listed in the order presented in 2B.

To identify true and false positives among the recovered clones, we knocked down each gene individually and retested their ability to impede doxorubicin induced apoptosis. Individual Open Biosystems shRNAs and a control were obtained from University of Minnesota RNAi core facility, packaged into lentivirus, infected into U2OS cells and selected with puromycin. These knockdown cell lines were then treated with 400 ng/ml doxorubicin and harvested 24 hours later for apoptosis analysis by propidium iodide (PI) staining measuring sub-G1 (apoptotic) DNA content (Figure 2A). Levels of apoptosis in control vector infected U2OS cells was designated at 100% and values expressed as % apoptosis between experimental verses control cell lines. We used paired T tests to determine which reductions in apoptosis induction were statistically significant. Nine knockdown cell lines (DCAF5, GPR45, UHRF2, MSH6, POLDIP2, HS3ST5, HORMAD2, FILIP1L, and PIGT) displayed a significant (p<0.05) reduction in apoptosis of 20% to 40% compared to control cells. The other three of the 12 candidates (MANF, UVRAG and ERI1) did not show significant reduction in doxorubicin induced apoptosis induction. Knockdown efficiency was measured by qPCR comparing target levels in targeted lines to levels in vector control U2OS cells. These results are displayed in Figure 2B as % remaining expression and range from 4% to 55% remaining expression.

**Figure 3:**
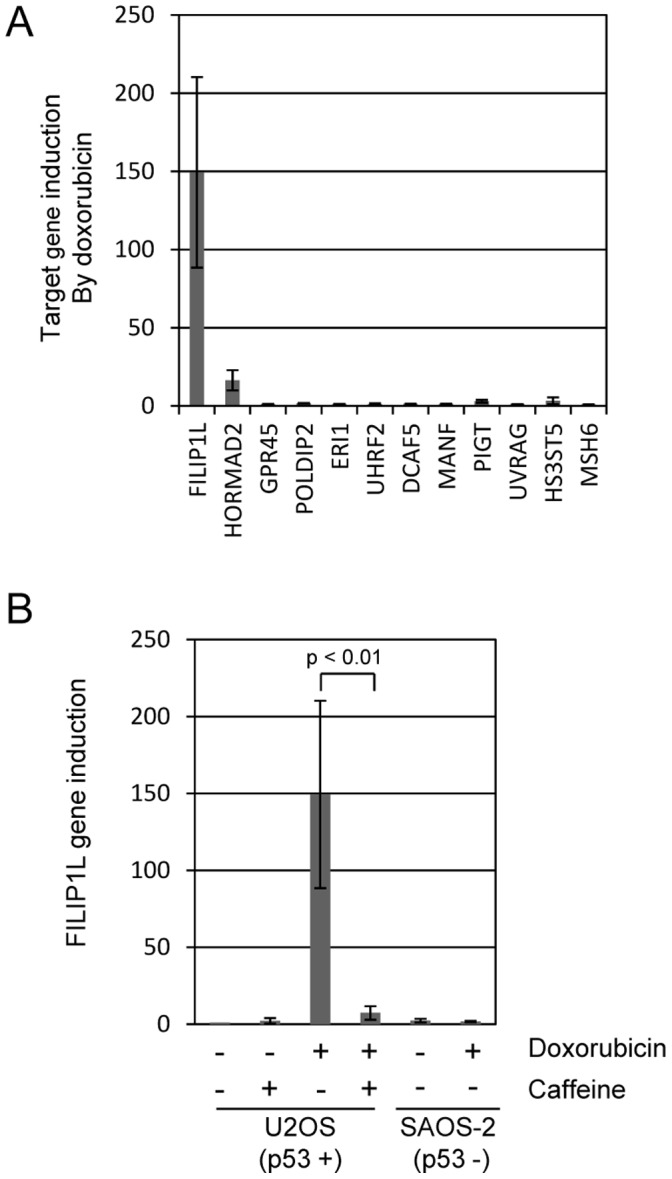
Doxorubicin treatment induces FILIP1L expression. (A) U2OS cells were treated with 200 ng/ml doxorubicin and mRNA isolated 24 hours later for qPCR analysis. The twelve genes identified in the shRNA screen were tested for induction by doxorubicin. Expression of most genes was unaffected by doxorubicin treatment. However, two genes, expression of FILIP1L and HORMAD2 were significantly induced by doxorubicin treatment, particularly FILIP1L which showed >200-fold induction. (B) FILIP1L induction by doxorubicin impaired following ATM/ATR inhibition in U2OS. Doxorubicin treatment induces DNA damage that activates the ATM and ATR kinases. Caffeine (4 mM) was used to inhibit ATM and ATR. FILIP1L induction by doxorubicin is reduced by over 90% by treatment with caffeine. SAOS-2 cells, which unlike U2OS do not contain wild-type p 53, fail to induce FILIP1L following doxorubicin treatment.

Damage of DNA by doxorubicin elicits many changes within the cell in an attempt either to deal with the damage or eliminate the cell. One effect is phosphorylation dependent recruitment of repair complexes to the damaged DNA. By contrast, DNA damage also alters gene expression patterns and induces target genes that function in DNA repair or apoptosis. We tested if treatment with doxorubicin altered expression of any of our candidate genes. U2OS cells were treated with 0 or 200 ng/ml doxorubicin and mRNA isolated 24 hours later for qPCR analysis. Gene expression levels were compared between drug and no drug treatment and displayed as fold-induction in Figure 3A. We determined that two of our candidate genes were induced by doxorubicin treatment: HORMA domain containing 2 (HORMAD2) and FILIP1L. FILIP1L was induced a striking 150-fold by doxorubicin treatment, whereas HORMAD2 was induced only around 10-fold. None of the other candidate genes showed differential expression following doxorubicin treatment. DNA damage caused by doxorubicin treatment activates the Ataxia telangiectasia mutated (ATM) and ataxia telangiectasia and Rad3-related (ATR) DNA repair checkpoint proteins [14]. We used caffeine to inhibit ATM/ATR activity following doxorubicin treatment to test the involvement of these signaling pathways in FILIP1L gene induction (Figure 3B). We determined that doxorubicin induction of FILIP1L was reduced around 90% by inhibiting ATM/ATR activity with caffeine treatment. These findings are consistent with the idea that DNA damage activates ATM/ATR and that one or both of these contribute to FILIP1L expression. The U2OS cell line contains a wild-type copy of p 53, a DNA damage responsive tumor suppressor gene. We tested if FILIP1L was induced in SAOS-2 cells which do not carry p 53. In sharp contrast to U2OS cells, doxorubicin treatment of SAOS-2 cells did not lead to any induction of FILIP1L. These findings are consistent with a model requiring ATM/ATR and p 53 for doxorubicin mediated induction of FILIP1L expression.

**Figure 4:**
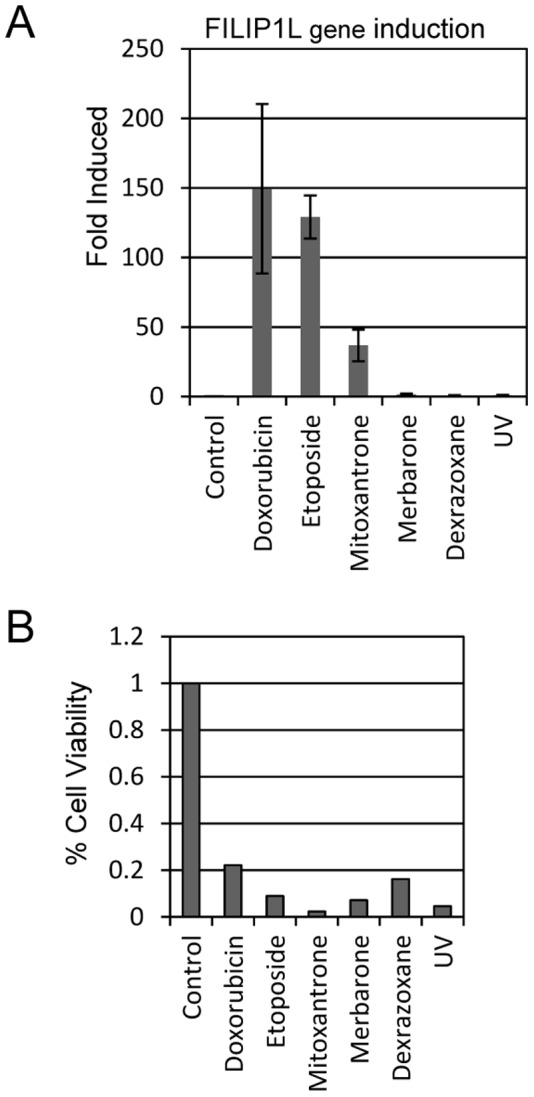
FILIP1L is induced by TOP2 poisons but not by catalytic inhibitors. (A) U2OS cells were treated with DMSO (Control), the TOP2 poisons doxorubicin (200 ng/ml), etoposide and mitoxantrone, or the TOP2 catalytic inhibitors merbarone or dexrazoxane. After 24 hours of treatment, mRNA was harvested from cells and FILIP1L expression levels were measured by qPCR analysis. (B) To allay concern that chosen drug dosages of the TOP2 inhibitors was too low to affect FILIP1L expression, we measured % cell viability after drug treatment. Treated cells were harvested after 24 hours and cell viability was measured using an Invitrogen Countess automated cell counter.

We tested additional drugs to determine whether these findings are specific to doxorubicin or apply to other TOP2 targeted agents. Drugs targeting topoisomerase II fall into two general categories, TOP2 poisons and TOP2 catalytic inhibitors. TOP2 poisons, which include doxorubicin, etoposide, and mitoxantrone, increase levels of TOP2-DNA complexes, and subsequent DNA lesions and strand breaks that elicit a DNA damage response. TOP2 catalytic inhibitors like merbarone, which impairs TOP2 DNA cleavage and dexrazoxane (ICRF-187), which inhibits TOP2 ATP hydrolysis, do not elevate TOP2-DNA covalent complexes. U2OS cells were treated with each drug for 24 hours before harvesting mRNA for qPCR analysis of FILIP1L levels. qPCR analysis indicated that treatment with any of the “DNA poisons” caused FILIP1L gene induction. For example, etoposide, like doxorubicin, led to over a 100-fold increased FILIP1L expression (Figure 4A). Similarly, mitoxantrone treatment increased FILIP1L treatment roughly 40-fold. However, neither merbarone nor dexrazoxane treatment caused increases in FILIP1L expression. These findings suggest that FILIP1L expression is responsive to several “TOP2 poisons” but not to two TOP2 catalytic inhibitors. We measured drug effects on U2OS cell viability to ensure that lack of FILIP1L expression was not due to insufficient dosages of merbarone and dexrazoxane. We observed a loss of viability of around 80% by each of the drug conditions tested, indicating that the TOP2 catalytic inhibitors cause cell death but do not induce FILIP1L expression (Figure 4B). UV irradiation also kills U2OS cells without inducing FILIP1L expression, indicating that not all types of DNA damage induce its expression.

**Figure 5:**
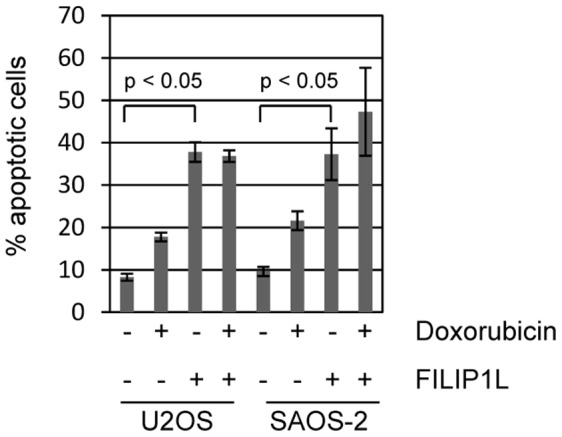
FILIP1L expression induces cell death. Ectopic expression of one of the identified genes, FILIP1L, caused significant induction of apoptosis on its own. U2OS and SAOS-2 cells were transfected with vector control (designated as “–” in the FILIP1L legend) or V5/His tagged FILIP1L expression plasmid. Cells were additionally treated with control or 200 ng/ml doxorubicin. Cells were harvested 24 hours after transfection and apoptotic cells were quantitated by measuring sub-G1 DNA content by propidium iodide staining. Apoptosis caused by FILIP1L expression in either cell type was not further augmented by treatment with doxorubicin.

We hypothesized that doxorubicin induces apoptosis in part through inducing FILIP1L expression. We tested the ability of ectopically expressed FILIP1L to induce apoptotic cell death. A V5/His-tagged FILIP1L or control plasmid were transfected into U2OS cells and treated with 0 or 200 ng/ml doxorubicin for 24 hours. Cells were harvested at 48 hours and analyzed for apoptotic DNA (sub-G1 content) by propidium iodide staining. Treatment with doxorubicin caused a modest 2-fold increase in apoptosis (Figure 5). However, FILIP1L expression led to around a 500% increase in apoptotic cell death. This death caused by FILIP1L was not further augmented by doxorubicin treatment. We also tested if FILIP1L expression was sufficient to induce apoptosis in SAOS-2 cells, which do not induce FILIP1L after treatment with doxorubicin (Figure 3B). Similar to experiments in U2OS cells, we observe around a 4-fold increase in apoptosis by ectopic FILIP1L expression, which is not significantly increased by additional treatment with doxorubicin.

**Figure 6:**
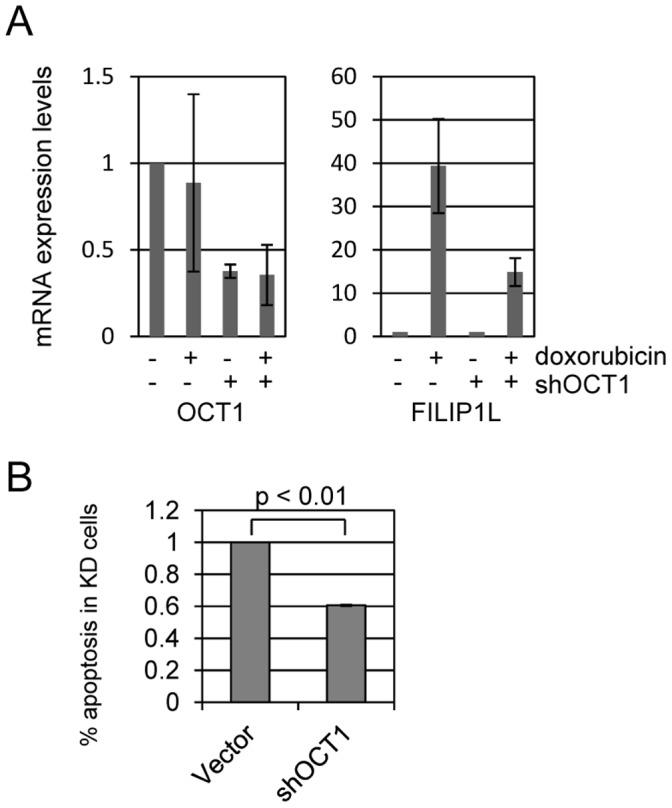
The transcription factor OCT1 mediates doxorubicin induced FILIP1L expression and apoptosis. OCT1, also called POU2F1 (POU domain, class 2, transcription factor 1), is a transcription factor that plays a role in the DNA damage response. We identified potential OCT1 binding sites in the FILIP1L promoter and tested if OCT1 is involved in Doxorubicin induced FILIP1L expression and apoptosis. (A) We targeted Oct1 for shRNA degradation in U2OS cells and used qPCR to verify 60% knockdown of target mRNA. Control and shOct1 cells were treated with 200 ng/ml doxorubicin and mRNA harvested 24 hours later for qPCR analysis. We determined that Oct1 mRNA levels are not affected by doxorubicin treatment. However, FILIP1L induction by doxorubicin is markedly reduced (∼65%) in shOct1 cells compared to control. (B) Control and shOct1 cells were treated with 400 ng/ml doxorubicin and measured for apoptosis at 24 hours. We found that Oct1 knockdown cells displayed 50% reduced doxorubicin induced apoptosis compared to control cells.

We analyzed the FILIP1L promoter for transcription factor binding sites that may potentiate doxorubicin induced expression using TFsearch online software (http://www.cbrc.jp/research/db/TFSEARCH.html) based on the TRANSFAC database [15]. This analysis revealed three potential OCT1 (POU2F1) binding sites in the FILIP1L promoter. OCT1 is a helix-turn-helix transcription factor that binds DNA as a monomer to an 8-bp sequence called the octamer motif (5′-ATGCAAAT-3′) [16]. The OCT1 transcription factor has been defined as a responder to DNA damage induced cellular stress [17]. OCT1 also contributes to the cellular response to ionizing radiation damage to DNA [18]. We tested the role of OCT1 in mediating doxorubicin induced apoptosis and FILIP1L expression. We targeted OCT1 for shRNA mediated degradation in U2OS cells and found that knockdown of OCT1 was around 60% effective (Figure 6A). We treated control and shOct1 cells with 0 or 200 ng/ml doxorubicin and measured POU2F1 (OCT1) and FILIP1L levels. OCT1 mRNA levels were not induced by treatment with doxorubicin (Fig. 6B). Knockdown of OCT1 did not affect baseline expression of FILIP1L. However, FILIP1L induction by doxorubicin was reduced around 65% by OCT1 knockdown. Furthermore, doxorubicin induction of apoptosis was reduced around 45% in shOct1 knockdown cells (Figure 6C). These findings indicate that doxorubicin activates the Oct1 transcription factor which in turn leads to expression of FILIP1L and causes apoptosis.

**Figure 7:**
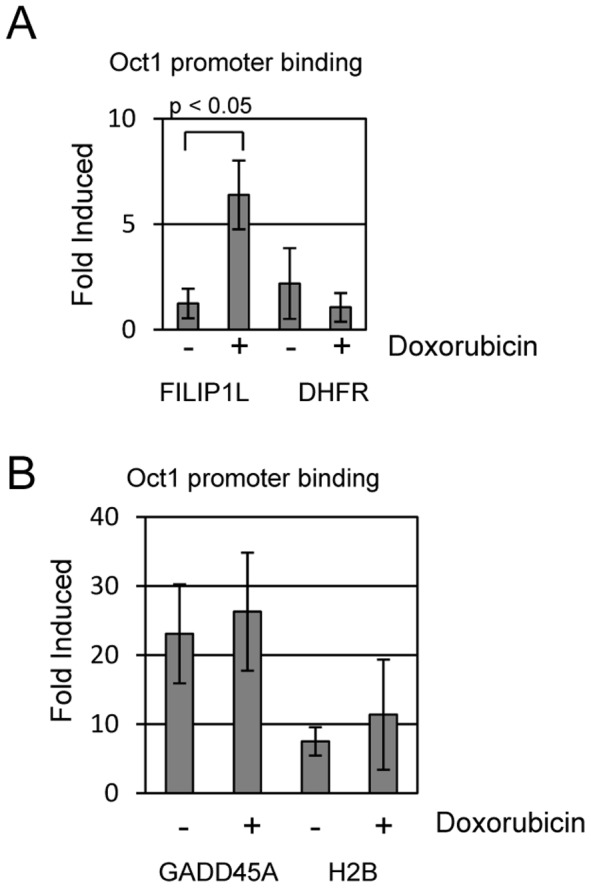
Doxorubicin induces OCT1 recruitment to the FILIP1L promoter. (A) We performed chromatin immunoprecipitation assays to determine if OCT1 binds to the FILIP1L promoter and if binding is influenced by doxorubicin. U2OS cells were treated with 0 or 400 ng/ml doxorubicin for 4 hours and then harvested. Chromatin was isolated and immunoprecipitated with control IgG or anti-Oct1 antisera. Units in this figure are “fold-induced” binding compared to binding we detect with IgG control (arbitrarily set to 1) used for the ChIP assay. ChIP analysis indicated that Oct1 does not bind to the FILIP1L promoter in unstressed conditions (no doxorubicin). However, treatment with doxorubicin resulted in a 6-fold induction of OCT1 binding to the FILIP1L promoter. OCT1 does not bind to the negative control dihydrofolate reductase (DHFR) promoter in either doxorubicin treated or untreated cells. (B) GADD45A and H2B are also OCT1 transcriptional target genes. We used ChIP to ask if doxorubicin induces OCT1 binding to these promoters. We determined that OCT1 binds to both promoters in the absence of doxorubicin, and treatment did not further stimulate OCT1 binding to either promoter.

We next tested if doxorubicin treatment causes Oct1 to be recruited to the FILIP1L promoter using chromatin immunoprecipitation. U2OS cells were treated with 0 or 400 ng/ml doxorubicin for 4 hours and then harvested for analysis. Chromatin was isolated from treated cells, sonicated, and immunoprecipitated with control IgG or Oct1 antibodies. We detect a 6-fold increase in Oct1 binding to the FILIP1L promoter after treatment with doxorubicin compared to binding observed in mock treated cells (Figure 7A). We also tested Oct1 binding to the GADD45A and H2B promoters, which previously showed increased Oct1 promoter binding following ionizing radiation DNA damage [18]. We observed higher basal Oct1 binding to both promoters in untreated cell. However, we did not observe increased Oct1 binding to either promoter following doxorubicin treatment (Figure 7B). These findings suggest that doxorubicin treatment causes recruitment of the Oct1 factor to the FILIP1L promoter and also induces FILIP1L expression in an Oct1 dependent manner. Other Oct1 regulated genes appear to show differential regulation by ionizing radiation compared with doxorubicin treatment, since doxorubicin had no effect on Oct1 recruitment to GADD45A or H2B.

## Discussion

In this study we used shRNA screening to identify genes that mediate the doxorubicin induced cell death program. Some of these identified targets appear to play a role in DNA metabolism and repair. For example MSH6 (mutS homolog 6) helps in the recognition of mismatched nucleotides prior to their repair [19]. POLDIP2 (polymerase, DNA-directed, delta interacting protein 2) encodes a protein that interacts with the delta p 50 subunit of DNA polymerase [20]. HORMAD2 (HORMA domain containing 2) contains a HORMA (for Hop1p, Rev7p and MAD2) domain that has been suggested to recognize chromatin states that result from DNA adducts, double stranded breaks or non-attachment to the spindle [21]. The other genes appeared diverse and not in the same category. For example, the FILIP1L protein contains an amino-terminal coiled-coil region and two leucine zipper motifs and shares similarity to bacterial SbcC, an ATPase DNA repair protein and exists as multiple isoforms in many cell types [22], [23]. However, the biochemical function of FILIP1L is unclear. UHRF2 ubiquitin-like with PHD and ring finger domains 2 is an E3 ubiquitin ligase, and DCAF5 (DDB1 and CUL4 associated factor 5) interacts with an E3 ubiquitin ligase [24], [25]. GPR45 is a G protein-coupled receptor [26]. HS3ST5 is a heparin sulfate (glucosamine) sulfotransferase [27]. The PIGT gene encodes a protein that is involved in glycosylphosphatidylinositol (GPI)-anchor biosynthesis.

**Figure 8:**
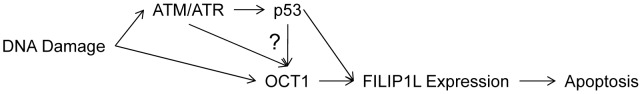
Model describing doxorubicin induction of FILIP1L and apoptosis. Our data suggests that doxorubicin treatment significantly activates FILIP1L expression and requires both OCT1 and ATM/ATR/p 53 activity for this expression. Ectopic expression of FILIP1L is sufficient for significant apoptosis induction. It is unclear how and if ATM/ATR and p 53 interact with Oct1, or if they function in via a parallel pathway to modulate FILIP1L expression. These findings suggest that FILIP1L expression may mediate doxorubicin induced apoptosis during chemotherapy and pose the hypothesis that cancers with down-regulated FILIP1L expression may display elevated doxorubicin resistance.

We focused these studies on the role of FILIP1L in mediating doxorubicin induced apoptosis. We demonstrated that doxorubicin treatment induces expression of FILIP1L in an ATM/ATR dependent manner. It also fails to be induced in SAOS-2 cells which lack the p 53 gene. Induction of FILIP1L and apoptotic cell death also requires the Oct1 transcription factor, and we show by ChIP that doxorubicin treatment causes Oct1 to relocate to the FILIP1L promoter. These findings indicate a model where doxorubicin treatment causes the Oct1 transcription factor to bind to the FILIP1L promoter to activate its expression followed by induction of apoptosis (Figure 8). They also suggest that loss of FILIP1L, which is observed in a variety of human tumors, might contribute to a poor response to doxorubicin.

The FILIP1L gene was originally identified as a gene down-regulated in ovarian cancer, or *DOC1*, compared to normal ovarian epithelial cells [28]. DOC1 was also identified as one of several genes observed to be elevated as prostate epithelial cells entered senescence and down-regulated in immortalized prostate cancer cell lines [29]. Down-regulation of FILIP1L in ovarian cancer has recently been linked to promoter methylation, although alternate modes of expression control likely also exist [30]. Kwon et. al. demonstrated that FILIP1L is highly induced in human umbilical vascular endothelial cells (HUVEC) by treatment with the anti-angiogenesis drug endostatin [31]. FILIP1L was one of the few genes identified from those studies that also displayed enhanced expression following 5-FU treatment, a different DNA damaging agent [22]. Furthermore, studies by Kwon et. al. demonstrated that ectopic expression of FILIP1L enforces an anti-proliferation block and also induces apoptosis in these cells [23]. At least one study has indicated that endostatin treatment sensitizes cancer cells to killing by doxorubicin, although it is unclear if this model involves increased FILIP1L expression [32]. The mechanism by which FILIP1L mediates apoptosis is unclear. FILIP1L contains a coiled-coil motif and two leucine zipper motifs that may facilitate protein-protein interactions. FILIP1L is remarkably similar to FILIP1, which can regulate cell motility by binding to filamin A and by inducing its degradation [33], [34]. FILIP1L appears to encode putative domains shared by chromosomal segregation proteins, and a domain found in cortactin binding proteins. It should be interesting to determine FILIP1L binding partners and potential localization to damaged DNA or chromosomes following its induction by various stresses.

FILIP1L expression is induced by DNA damage by the “TOP2 poisons” doxorubicin, etoposide and mitoxantrone, but not by TOP2 catalytic inhibition by merbarone or dexrazoxane. Doxorubicin and mitoxantrone are anthracycline DNA intercalating agents that trap TOP2 in covalent complexes with DNA. These complexes cause DNA lesions and strand breaks and as such have been termed DNA poisons. Etoposide is non- intercalating anthracycline poison that is believed to block DNA re-ligation following DNA cleavage. While etoposide inhibits TOP2 function, it also generates significant DNA damage through strand break generation similar to doxorubicin. We observed that treatment with each of these DNA damaging compounds causes induction of FILIP1L. The particular type of DNA damage is important, as we demonstrated that UV irradiation does not induce FILIP1L expression. Other compounds inhibit TOP2 catalytic activity without inducing TOP2 covalent complexes with DNA. These agents are believed to kill cells since TOP2 activity is essential for DNA replication and cell division. For example, merbarone prevents TOP2 mediated DNA cleavage, and dexrazoxane (ICRF-187) inhibits ATP hydrolysis and maintains the TOP2 structure as a closed clamp. Both of these drugs kill U2OS cells without inducing FILIP1L. These findings suggest that DNA strand breaks, but not TOP2 catalytic inhibition or UV mediated DNA crosslinking, cause FILIP1L expression.

We identified several components of the signaling pathway between doxorubicin and FILIP1L expression. First, FILIP1L expression depended on the activity of ATM (ataxia-telangiectasia, mutated)/ATR (ATM and Rad3-related) and was inhibited by treatment with caffeine. ATM and ATR are Phosphatidyl-inositol-3 kinase (PI3K) family members that respond to DNA damage signals. ATM responds primarily to double-strand breaks induced by ionizing radiation whereas ATR also responds to DNA damage caused by ultraviolet light and stalled replication forks [14].

We also determined that the transcription factor Oct1, (a product of the *POU2F1* gene) is important for FILIP1L induction by doxorubicin. Oct1 induces stress responsive target genes following genotoxic damage [17]. Kang et. al. demonstrated that ionizing radiation alters phosphorylation and target gene localization of Oct1 and causes it to be recruited to the *H2B* and *Gadd45a* gene promoters [18]. Our results build on these findings by demonstrating that DNA damage caused by doxorubicin also causes Oct1 to become relocalized to the FILIP1L promoter and induce its expression, thereby facilitating cell death. The extent that FILIP1L expression mediates doxorubicin induced killing *in vivo*, or that FILIP1L loss in human tumors impedes doxorubicin based chemotherapy will be important to assess.

## Materials and Methods

### shRNA Screen

PLAT-A cells were previously obtained from T. Kitamura [35]. U2OS and SAOS-2 cells were obtained from ATCC. The human shRNAmir library (Open Biosystems) was divided into 30 pools with 1000 shRNAs per pool [12]. We screened eight of the thirty pools, or around 26% of the entire library. Pooled shRNA plasmids were packaged into retrovirus using PLAT-A packaging cell lines and infected into the U2OS human osteosarcoma cell line. Approximately 2×10^7^ U2OS cells were infected by library retroviral shRNAs at a multiplicity of infection of 0.5. Stably transfected cells were generated by puromycin selection. Cells were treated with 225 ng/ml doxorubicin for 5 days. Cells that survived doxorubicin treatment were pooled, genomic DNA recovered from them, and the shRNAs identified by PCR amplification, shotgun cloning into TOPO TA vector (Invitrogen) followed by sequence analysis. We sequenced a total of 1500 clones and have listed recurring clones in Figure 1B. 1488 single clones were identified and are not listed. A total of 8 of the 30 pools (around 26% of the entire library) were screened in these analyses.

### Cell Culture and DNA Plasmids

U2OS (human osteosarcoma) cells were cultured in Dulbecco’s Modified Eagle Medium (DMEM) media containing 10% fetal calf serum. Floating and adherent cells were harvested at 40 hours post-infection, and assayed for apoptosis using propidium Iodide (PI) staining to measure sub-G1 DNA content (Sigma). Cell viability was measured using an Invitrogen Countess Automated Cell Counter. Individual Open Biosystems shRNA plasmids were obtained from the University of Minnesota RNAi core facility. We obtained a V5/His tagged FILIP1L expression plasmid from Open Biosystems. Caffeine, doxorubicin, etoposide, mitoxantrone, dexrazoxane, and merbarone were obtained from Sigma. Caffeine was used at a concentration of 4 mM. Doxorubicin was used at 200 ng/ml for gene expression studies and 400 ng/ml for apoptosis induction. Etoposide was used at 20 µM, mitoxantrone (0.5 µM), merbarone (100 µM), and dexrazoxane (100 µM). For UV irradiation, medium was removed from U2OS cells and the cells were irradiated in a UV Stratalinker (Stratagene) with 120 J/m2 and culture medium was then restored.

### RNA Isolation & Real-time PCR

We isolated RNA from cells using QIAGEN QIAshredder and RNeasy Midi Kits. We used the QuantiTect SYBR Green RT-PCR kit from QIAGEN according to manufacturer’s specifications for our quantitative real-time PCR. Each experimental condition used 100 ng of RNA for reverse transcription and RT-PCR and was performed in triplicate and normalized against GAPDH expression levels. Analysis was done with a StepOnePlus real-time PCR system (Applied Biosystem) according to the manufacturer’s protocol. Error bars represent SD and experiments represent at least three independent replicates.

The following primers were used for real-time PCR.

FILIP1L (5′: GCATTCTGGAGGGAGAACTG; 3′: TAGATGTCCTCCTGCCAAGG),

HORMAD2 (5′: CTGCTCAGCTTTCTCACTGC; 3′: GGAAACAGGCCCCTTAGGTA)

GPR45 (5′: ATTTCTGTCCCAGCTCCAAG; 3′: GGCCTCTGGTACACGATGAT)

POLDIP2 (5′: GGTCGGGCTCTGTGTCAG; 3′: TCTCCAACACTTTGCCCTCT)

ERI1 (5′: GCATGGAGGATCCACAGAGT; 3′: AAGTCACTCGCACTGGAGGT)

UHRF2 (5′: TTGCTGCTGATGAAGACGTT; 3′: TTCTGCATCAAACCAGAATCC)

DCAF5 (5′: GTCAGTGGTGGGCTTCTTGT; 3′: GAGTGGATGGCTTGTTCCAT)

MANF (5′: GCAAGAGGCAAAGAGAATCG; 3′: GCTCACATATCTGGCTGTCCT)

PIGT (5′: GGGAGGAACTTGTCATCACC; 3′: CAGTATCGGGTCCTCCAAAA)

UVRAG (5′: GCGGTGTCAAGTTGCCTAAT; 3′: AAGCACCCACTGATCCAGAC)

HS3ST5 (5′: GAGGGCCATGCTATTCAAAC; 3′: AGCAGGCCACGCTTAAACT)

MSH6 (5′: AAGGCGAAGAACCTCAACG; 3′: TGTTGGGCTGTCATCAAAAA)

### Chromatin Immunoprecipitation

Sonicated genomic extracts were prepared from U2OS cells following the protocol described by Bomsztyk [36]. Two 15-cm plates of cells treated with 0 or 400 ng/ml doxorubicin (4 hours) and then fixed with formaldehyde for 15 minutes at room temperature and then quenched with 125 mM glycine for 5 minutes. Cells were collected, washed, and resuspended with 300 µl lysis buffer (1% SDS, 10 mM EDTA, 50 mM Tris, pH 8.0.), diluted 10x with dilution buffer (1% Triton X-100, 150 mM NaCl, 2 mM EDTA, 20 mM Tris, pH 8.1). The DNA pellet was resuspended in IP buffer and sonicated with a Branson model 450 Sonicator to an approximate length of 550 nucleotides. Immunoprecipitations were set up using mock IP or combined anti-Oct1 (cat #s A301-716A and A301-717A, Bethyl Laboratories INC) antibodies at 4 degrees overnight and then treated with protein A-agarose beads for 60 minutes. Complexes were centrifuged and washed three times with cold IP buffer (150 mM NaCl, 5 mM EDTA, 0.5% NP40, 1% Triton-X 100, 50 mM Tris pH 7.5). 100 microliters of 10% Chelex 100 (Bio-Rad) was added to precipitated DNA samples and then ethanol precipitated. The following primers were used for ChIP:

FILIP1L (5′: TTTCATCTGGCCTTTCCATC 3′: GAAGTCTGGGGTTTTGTGGA)

GADD45(5′: CTCCTCTCAACCTGACTCCAGGAG 3′: TCCGGGGTTATCCTGCCAAC)

H2B (5′: GGATTTGCGAATCCTGATTGGGCA 3′: AGCACTGTGTAGCTATAAAGCGCC)
